# Membrane Technologies for Microplastics Removal from Wastewater: Recent Progress, Fouling Mechanisms, and Future Perspectives

**DOI:** 10.3390/membranes16060190

**Published:** 2026-06-01

**Authors:** Soon Onn Lai, Mohammed J. K. Bashir, Choon Aun Ng, Kok Chung Chong, Heng Keong Kam, Riza P. Gumaling, Lin-Chi Wang

**Affiliations:** 1 Lee Kong Chian Faculty of Engineering and Science, Universiti Tunku Abdul Rahman, Sungai Long Campus, Jalan Sungai Long, Cheras, Kajang 43000, Selangor, Malaysia; 2 Centre for Advanced and Sustainable Materials Research, Universiti Tunku Abdul Rahman, Sungai Long Campus, Jalan Sungai Long, Cheras, Kajang 43000, Selangor, Malaysia; 3 School of Engineering and Technology, Central Queensland University, 120 Spencer St., Melbourne, VIC 3000, Australia; 4 Faculty of Engineering and Green Technology, Universiti Tunku Abdul Rahman, Jalan Universiti, Bandar Barat, Kampar 31900, Perak, Malaysia; 5 Environmental Engineering Department, College of Engineering, Western Mindanao State University, Baliwasan, Zamboanga City 7000, Zamboanga Del Sur, Philippines; 6 Department of Marine Environmental Engineering, National Kaohsiung University of Science and Technology, Kaohsiung 811213, Taiwan

**Keywords:** microplastics, membrane fouling mechanisms, wastewater reclamation, smart and functionalized membranes, artificial intelligence (AI) optimization

## Abstract

Microplastics have recently emerged as a widespread contaminant in wastewater, posing severe risks to the environment and human health due to their potential bioaccumulation and toxicity. Conventional wastewater treatment processes are generally inadequate for the complete removal of microplastics due to their modest scale. Interest has been garnered from academia and industry regarding their separation from wastewater. This review covers recent advances in the application of membrane processes for the removal of microplastics from wastewater. The principles of membrane separation, removal efficiency, and operational challenges are critically evaluated, along with the potential of the hybrid membrane systems. In the next section, the fouling mechanism induced by microplastics and their interaction with foulants, as well as cleaning and anti-fouling strategies, are discussed. Finally, future perspectives focus on the current unresolved research gaps, including the integration of digital monitoring and artificial intelligence-assisted optimization of membrane technology for microplastic removal. By consolidating current knowledge and identifying pathways for innovation, this review underscores the pivotal role of membranes in mitigating plastic pollution and advancing sustainable wastewater management.

## 1. Introduction

The use of plastic materials has risen to one of the most challenging environmental issues of the 21st century. The demand for plastic continues to rise, and the fragmentation of the used plastic waste leads to degradation and the presence of microplastics (MPs) (particles < 5 mm) across diverse ecosystems [1]. Wastewater treatment plants in the world were conventionally designed to remove organic matter and nutrient discharge from residential, commercial, and industrial activities. These conventional treatment plants generally undergo primary sedimentation and activated sludge, which can remove most of the solids, but it is often inadequate for the complete retention of smaller MPs, leading to the discharge of these particles into aquatic environments annually [2,3].

Microplastics are now classified as “emerging contaminants” in academia, attributed to their persistence and unique physicochemical properties [4]. Different from the traditional soluble pollutants, microplastics are a solid containment that possess high surface area to volume ratio, allowing them to act as a carrier for heavy metals and organic pollutants. The high concentration of microplastics in the wastewater not only presents as a physical pollutant but also alters the chemical and biological dynamics of the wastewater content, leading to a more complicated treatment technique [5,6].

The microplastics can be found in both primary and secondary sources. The municipal effluents consist of secondary microplastics derived from the synthetic microfiber from laundry and small fragments from the degradation of consumer products such as plastic bottles or food packaging [7]. On the other hand, industrial effluent comprises primary sources of microplastics, particularly discharge from textile, plastic, and recycling industries. These industries contribute microplastics mostly in the nurdles, polymer bead and fibers. Recent literature studies also suggested that the urban runoff and tire wear particles also infiltrate sewer systems, further diversifying the polymer profiles [8].

The ecological impact of the microplastic is attributed to the potential of their bioaccumulation and trophic transfer. Due to their small size, the microplastic particles are readily ingested by the aquatic organisms, where these particles will cause the inflammatory responses of the organism and a reduction in the efficiency of the reproduction system [9,10]. Subsequently, these microplastic possess the ability to penetrate through the circulatory system of the organism and increase the level of toxicity in the body [11].

Given the limitations of the conventional system, membrane technologies such as microfiltration (MF), ultrafiltration (UF), nanofiltration (NF), and reverse osmosis (RO) have emerged as the potential solution for the high efficiency microplastic removal [12,13]. Traditionally, these processes rely on polymer-based membranes due to their mechanical robustness and cost-effectiveness; however, they are fundamentally limited by an intrinsic trade-off between permeance and selectivity. In these dense polymer networks, non-uniform transport pathways often lead to reduced efficiency, while the inherent tendency of polymer surfaces to adsorb organic foulants undermines long-term operational stability [14]. To overcome these constraints, the field has transitioned toward next-generation hybrid membrane architectures that incorporate two-dimensional (2D) nanomaterials into processable polymer matrices. Among these materials, MXenes, a family of 2D transition metal carbides and nitrides have gained significant attention due to their high hydrophilicity, tunable interlayer spacing, and excellent surface reactivity [15]. Unlike traditional polymeric membranes, MXene-polymer hybrid structures, such as thin-film nanocomposites (TFNs) and mixed matrix membranes (MMMs), utilize strong coordinative interactions and hydrogen bonding to regulate the polymer chain packing and interfacial polymerization process [16]. This chemical integration allows for the creation of fast-transport nanochannels that enhance water flux while maintaining high rejection rates, effectively decoupling water transport efficiency from pore size enlargement. Furthermore, the incorporation of MXenes provides superior surface hydrophilicity and long-term antifouling characteristics, addressing the stability issues prevalent in pristine polymer systems. Nevertheless, the actual microplastic removal in the industrial application using a membrane might be hindered due to the high energy demands and complexity of the fouling mechanism in the membrane system. Therefore, this review aims to provide a consolidation of the current development of membrane technology in microplastic removal and identification of innovative pathways to progress.

## 2. Membrane Processes for Microplastic Removal

Over the past decade, increasing evidence has demonstrated that membrane filtration can achieve removal efficiencies exceeding 90–99% for MPs when appropriately selected and integrated into treatment trains [17]. As a result, membrane processes are now widely applied in municipal wastewater treatment plants (WWTPs), industrial wastewater treatment, drinking water treatment plants (DWTPs), and advanced water reuse systems [18]. Membrane technologies such as microfiltration (MF), ultrafiltration (UF), nanofiltration (NF), and reverse osmosis (RO) differ in pore size, operating pressure, selectivity, and fouling behavior. These differences determine their suitability for removing microplastics of varying sizes, shapes, and polymer compositions. Moreover, hybrid configurations that integrate membranes with biological treatment, coagulation, or advanced oxidation processes have demonstrated enhanced performance and operational resilience [19]. The following sections provide a comprehensive discussion of individual membrane processes, their mechanisms of microplastic removal, reported efficiencies in different wastewater matrices, real-world case studies, and associated operational challenges.

### 2.1. Microfiltration (MF)

Microfiltration membranes typically possess pore sizes ranging from 0.1 to 10 µm, making them particularly suitable for removing relatively large microplastic particles commonly present in municipal and industrial wastewater [20]. The primary mechanism governing microplastic removal in MF systems is size exclusion, whereby particles larger than the membrane pores are physically prevented from passing through the membrane matrix. In addition to size-based rejection, hydrodynamic interactions between suspended particles and the membrane surface, as well as particle deposition under cross-flow conditions, contribute to enhanced microplastic retention. These mechanisms are especially effective for fibrous and irregularly shaped microplastics, which are more prone to entanglement and surface accumulation [21]. Cross-flow filtration is generally considered superior to dead-end filtration for the removal of microplastics and nanoplastics from water and wastewater due to its enhanced fouling control, operational stability, and longer membrane lifespan. In cross-flow filtration, the feed stream flows tangentially across the membrane surface, generating shear forces that continuously sweep away accumulated particles and reduce the formation of a dense fouling layer. In contrast, dead-end filtration forces the entire feed flow perpendicular to the membrane surface, causing retained particles to accumulate rapidly and form a compact cake layer. While this cake layer may initially improve particle rejection, it leads to rapid flux decline and frequent membrane clogging, particularly in wastewater streams with high suspended solids content.

During prolonged MF operation, the accumulation of suspended solids, organic matter, and retained microplastics on the membrane surface leads to the formation of a cake layer. Although membrane fouling is generally considered a drawback due to its negative impact on permeate flux, the presence of a cake layer can improve microplastic removal by acting as a secondary filtration barrier [22]. This layer effectively reduces the apparent pore size of the membrane, thereby enhancing the rejection of smaller microplastic particles that might otherwise pass through. Furthermore, electrostatic interactions between charged microplastic surfaces and membrane materials may influence retention efficiency, particularly in complex wastewater matrices containing dissolved organic matter and inorganic ions [23,24].

Numerous studies have reported high microplastic removal efficiencies using MF membranes in municipal wastewater treatment applications. Removal rates exceeding 90% have been observed for microplastics larger than 10 µm in secondary effluents, with fibrous and fragment-shaped particles being particularly well retained [25]. In industrial wastewater treatment, MF has demonstrated similarly high efficiencies, although performance may vary depending on wastewater composition and suspended solids concentration [26]. Despite its effectiveness for larger particles, MF is limited in its ability to retain small microplastics and nanoplastics due to intrinsic pore size constraints. As a result, MF is often employed as a preliminary or pretreatment step before more selective membrane processes such as UF or NF. This staged approach helps reduce fouling loads on downstream membranes while improving overall microplastic removal efficiency [27]. Yet, MF offers a superior alternative for traditional filtration methods because of its higher and more constant quality, lower energy use, and the possibility of continuous operation.

### 2.2. Ultrafiltration (NF)

Ultrafiltration membranes provide smaller pore sizes, typically in the range of 1–100 nm, enabling enhanced removal of smaller microplastics and a significant fraction of nanoplastics [28]. Similar to MF, size exclusion remains the dominant removal mechanism; however, the reduced pore size of UF membranes allows for more effective retention of particles approaching the sub-micron scale. Electrostatic interactions between charged plastic particles and membrane surfaces may further enhance rejection under certain conditions [29]. Unlike conventional filtration, NF rejection is governed by more than just physical dimensions; it is heavily influenced by the surface charge of the membrane, the morphology of solute ions, and localized dielectric properties. For charged or dissociable particles, rejection is significantly dictated by the electrical properties of the membrane surface [30]. Many contaminants such as MPs are retained through electrostatic repulsion between the charged solute and the charged membrane surface. The effectiveness of this mechanism is often dependent on the pH of the wastewater [31]. For example, the retention of easily dissociated compounds like ibuprofen is more significantly affected by electrostatic repulsion as pH fluctuations change the charge state of the molecules and the membrane. At the nanoscale, dielectric effect enhancement becomes a dominant factor in solute rejection. This occurs due to the interaction between ions and the polarized environment at the membrane-solution interface. These effects create an additional energy barrier that hinders the entry of ions into the membrane pores, regardless of their physical size, thereby enhancing the overall rejection of charged ultra-small particles.

Experimental studies have demonstrated that UF membranes outperform MF systems in retaining nanoplastics, particularly polystyrene particles with diameters below 500 nm. While MF membranes often allow partial passage of these particles, UF membranes have shown rejection efficiencies exceeding 90% under optimized laboratory conditions [32]. However, the finer pore structure of UF membranes makes them more susceptible to fouling, especially internal pore blocking caused by organic matter and colloidal particles, which is why MF usually acted in first filtering [33].

Laboratory-scale studies have demonstrated that UF membranes outperform MF systems in retaining nanoplastics, particularly polystyrene particles with diameters below 500 nm. While MF membranes often permit partial passage of these particles, UF membranes have achieved rejection efficiencies exceeding 90% under optimized experimental conditions. In full-scale municipal WWTPs, UF is commonly implemented as a tertiary treatment process following biological treatment. Field investigations have reported overall microplastic removal efficiencies ranging from approximately 80% to nearly 100% when UF is integrated into conventional treatment trains, representing a substantial improvement over traditional tertiary treatments such as rapid sand filtration or disc filtration [34].

UF membranes have also been successfully applied in industrial wastewater treatment, including effluents from textile manufacturing, plastic processing, and food industries. In these applications, UF provides reliable removal of fine microplastics while producing high-quality effluent suitable for reuse or safe discharge. However, the finer pore structure of UF membranes makes them more susceptible to fouling, particularly internal pore blocking caused by organic matter, colloids, and biofilms [35]. As a result, effective fouling management strategies, including regular backwashing, air scouring, and periodic chemical cleaning, are essential to ensure long-term operational stability and economic viability [36].

### 2.3. Nanofiltration (NF) and Reverse Osmosis (RO)

Nanofiltration and reverse osmosis represent the most selective membrane processes available for the removal of microplastics and nanoplastics from water and wastewater. NF membranes typically exhibit effective pore sizes in the range of 1–10 nm, enabling them to retain nanoplastics, dissolved organic matter, and multivalent ions through a combination of size exclusion and electrostatic repulsion. In contrast, RO membranes are essentially non-porous and operate based on a solution–diffusion mechanism, providing near-complete rejection of particulate and dissolved contaminants, including nanoplastics and plastic-associated additives [37].

Research has consistently shown that NF membranes can achieve nanoplastic removal efficiencies exceeding 90% when operated under appropriate transmembrane pressures and water chemistry conditions. RO systems, on the other hand, demonstrate the highest removal efficiencies, often exceeding 99%, making them particularly suitable for applications requiring stringent effluent quality, such as drinking water production and potable water reuse. Due to their exceptional barrier properties, RO membranes are widely regarded as the most reliable technology for preventing microplastic contamination in finished drinking water [38].

Despite their superior performance, NF and RO systems face notable operational challenges. Both processes require relatively high operating pressures, particularly RO, which leads to increased energy consumption and higher operational costs. Additionally, membrane fouling and concentrate management remain critical concerns, as retained microplastics, salts, and other contaminants accumulate in the reject stream and require appropriate treatment or disposal. These limitations highlight the importance of effective pretreatment and integrated system design to maximize performance while minimizing environmental and economic impacts [39]. A comprehensive comparison of the technical specifications, removal mechanisms, and operational performance of these various membrane processes for micro- and nanoplastic mitigation is summarized in Table 1.

### 2.4. Hybrid and Integrated Systems

Hybrid and integrated treatment systems represent an advanced approach to microplastic removal by combining membrane filtration with biological, chemical, or physical processes to overcome the limitations of standalone membrane technologies. While individual membrane processes such as MF, UF, NF, and RO are highly effective at physically retaining microplastics, their long-term performance is often constrained by membrane fouling, concentrate management, and operational instability. By integrating complementary treatment processes, hybrid systems enhance removal efficiency, improve membrane durability, and provide greater resilience when treating complex wastewater matrices containing organic matter, colloids, and variable microplastic loads [40].

One of the most widely implemented hybrid configurations is the membrane bioreactor (MBR), which integrates conventional activated sludge treatment with MF or UF membranes. In MBR systems, biological degradation of organic pollutants occurs simultaneously with physical separation by the membrane, allowing microplastics to be retained within the bioreactor while treated water passes through the membrane barrier [41]. This dual mechanism significantly improves microplastic removal compared to conventional activated sludge systems, where a substantial fraction of microplastics may escape with the final effluent. Field and laboratory studies have consistently reported microplastic removal efficiencies exceeding 99% in MBR systems, with particularly high retention of fibrous and fragment-shaped particles [42]. In addition to improved removal performance, MBRs operate at higher mixed liquor suspended solids concentrations, which further promotes the entrapment of microplastics within sludge flocs, thereby reducing their release into receiving water bodies.

Advanced oxidation processes (AOPs), when coupled with membrane filtration, provide an additional layer of treatment by targeting concentrated polymeric contaminants in membrane retentate streams [43]. Processes such as ozonation, UV/H_2_O_2_, and photocatalysis have demonstrated potential for partially degrading microplastics and polymer additives into smaller, more biodegradable compounds. When integrated with NF or RO systems, AOPs can be applied to the concentrate stream to reduce secondary pollution risks associated with microplastic accumulation [44]. Although complete mineralization of plastics remains challenging, the combination of physical separation and chemical degradation offers a promising strategy for managing retained microplastics in high-recovery membrane systems [45].

Multi-stage membrane treatment trains, such as MF–UF–NF or MF–UF–RO configurations, represent another important class of integrated systems designed to achieve graded microplastic removal [46]. In these configurations, MF serves as a pretreatment step to remove large microplastics and suspended solids, thereby protecting downstream membranes from excessive fouling. UF then targets smaller microplastics and nanoplastics, while NF or RO provides final polishing by removing the finest particles and dissolved polymeric compounds. This staged approach balances removal performance with energy efficiency and operational cost, making it particularly suitable for advanced wastewater treatment and water reuse applications. Studies have shown that such multi-barrier systems can achieve near-complete microplastic removal while maintaining stable long-term operation [47].

Despite their superior performance, hybrid and integrated membrane systems are associated with higher capital investment and increased operational complexity compared to single-process treatments. The need for additional infrastructure, chemical dosing, process control, and skilled operation can increase overall costs. However, these disadvantages are often outweighed by the synergistic benefits of improved effluent quality, reduced environmental risk, and enhanced system robustness. As regulatory pressure on microplastic emissions increases and water reuse becomes more widespread, hybrid membrane systems are expected to play an increasingly critical role in sustainable water and wastewater management. By integrating biological, chemical, and physical treatment mechanisms, these systems offer adaptable and resilient solutions for mitigating microplastic pollution across diverse water matrices and operational conditions [48].

## 3. Membrane Fouling Induced by Microplastics

### 3.1. Interaction Between MPs and Organic/Inorganic Foulants

Microplastics function as significant environmental carriers because of their small particle sizes and massive specific surface areas. These particles do not just drift alone but instead physically adsorb or chemically react with organic and inorganic pollutants to create complex binary pollutants [49,50]. This interaction is primarily driven by non-covalent forces such as hydrophobic attraction and electrostatic interactions alongside covalent interactions like metal–organic complexation. The surface potential of microplastics is a critical factor in how these composite contaminants form and eventually impact treatment systems [51,52]. In natural aquatic environments, there is often a positive correlation between the number of microplastics found in an organism and the concentration of organic or inorganic pollutants in its tissue.

When microplastics interact with organic foulants like humic acid or dissolved organic matter, the process is largely dictated by interactions and hydrophobic forces. Studies show that humic-like fractions of organic matter bind particularly well to microplastics because of their high aromaticity and phenolic content [53,54]. These interactions are often spontaneous and involve specific functional groups such as carboxylic acid and aromatic rings. Interestingly, the presence of microplastics can promote the adsorption of heavy metals onto organic matter. For instance, lead can first adsorb onto the surface of polystyrene microplastics through electrostatic action and then indirectly bind to humic acid as the plastic and organic matter link together [55]. This creates a composite pollutant that increases the total amount of metal held by the organic matter and alters its environmental behavior.

The interaction with inorganic foulants like heavy metals and mineral scalants is further influenced by the physical state of the microplastic [56]. Microplastics can enrich metals such as copper, lead, and chromium from their surroundings. This capacity changes significantly as microplastics age due to UV radiation or microbial activity. The aging process creates physical cracks and pits on the plastic surface, which increases the available surface area for foulants to attach. Photo-oxidation also leads to the formation of oxygen-containing functional groups like carbonyl and hydroxyl groups. These changes typically increase the negative surface charge of the microplastics and enhance their ability to bind with inorganic cations [57].

In the context of membrane filtration, these interactions lead to combined fouling where microplastics and other pollutants accumulate together [58,59]. The structure of the resulting fouling layer is determined by how well the particles and organic biopolymers stick together. If microplastics interact strongly with organic matter, they can form agglomerates that build a porous backbone cake layer on the membrane surface [60]. This type of layer is often more filterable than a continuous gel layer. However, if the interaction is weak, the organic polymers remain free to form a dense and nearly impermeable gel that fills the pores of the cake layer and drastically increases filtration resistance. Some inorganic particles, like silica, can aggravate this issue, while others, like ferric oxides, may alleviate it by promoting better adsorption of the organic foulants.

The specific sequence in which these foulants bind is also a key part of the interaction mechanism. In systems where microplastics coexist with heavy metals and organic matter, the metals often bind to humic-like fractions before fulvic-like fractions [61]. Chemical analysis suggests that the structural changes in organic matter upon binding follow a specific order, starting with carboxylic acids and moving through various other groups. The amount of microplastic added to a system directly affects both the degree of binding and the order in which functional groups react. Ultimately, these complex interactions determine whether the fouling layer will be primarily gel-like or cake-like, which dictates the overall performance of the treatment process [62].

### 3.2. Impact on Membrane Permeability and Selectivity

In industrial separation and water treatment, membranes serve as a critical barrier, yet the introduction of microplastics into feed streams significantly challenges operational efficiency [63,64]. The impact of such fouling is primarily measured through the degradation of two vital metrics: permeability and selectivity. Permeability represents the overall throughput, the speed at which molecules traverse the material, while selectivity defines the efficiency of the separation itself. For a membrane to be considered ideal, it must remain both highly permeable and highly selective, but the presence of microplastics often forces an unwanted compromise between these two qualities [65].

The accumulation of microplastic particles on the membrane surface or within its pores initiates a cascade of performance issues. This particulate and organic fouling often results in the formation of a “cake layer,” a physical barrier that restricts the flow of water or gas [66]. As these insoluble particles gather, they create a bottleneck that drastically reduces permeability. To maintain a steady flux in the presence of such a layer, higher operating pressures are required, which, in turn, increases energy consumption and places significant structural stress on the membrane material [67]. Over time, this cake layer can compact, further impeding flow and demanding even higher pressure to drive molecular transport.

Beyond the visible surface blockage, microplastics influence the internal physics of the polymer matrix. In many amorphous membranes, transport depends on Free Volume Elements (FVEs), which are small, unoccupied void spaces between polymer chains resulting from inefficient packing [68]. These FVEs facilitate the diffusion of desired molecules while hindering larger ones. When fouling occurs, the resulting pressure and physical entrapment can induce structural deformation [69]. As the membrane experiences this deformation, the sizes and distribution of its FVEs change. Molecular Dynamics (MD) simulations have demonstrated that such changes typically cause FVEs to become larger or more widely distributed, which directly impacts the membrane’s transport properties [70].

This shift in the internal architecture strikes at the heart of the “permeability-selectivity trade-off,” a concept frequently illustrated by the Robeson upper bound [71]. This bound describes an inherent limit where increased permeability often leads to decreased selectivity. Microplastic fouling pushes performance further from this ideal. By blocking the most efficient transport pathways or causing the pore structure to widen through deformation, the membrane loses its ability to perform precise size-based sieving [72]. Consequently, unwanted solutes may find pathways through the altered structure, leading to inconsistent product quality and lower separation efficiency.

The long-term consequences of these impacts extend to the lifespan of the membrane module itself. Frequent chemical cleaning is often necessary to strip away the resilient “sticky layer” formed by microplastics and associated extracellular polymeric substances [73]. However, repeated exposure to harsh cleaning agents eventually degrades the polymer chains, further compromising the mechanical strength and the robust distribution of FVEs required for effective separation [74]. While advanced materials, such as thermally rearranged (TR) polymers, exhibit more stable FVE distributions under stress, the persistent presence of microplastics remains a fundamental hurdle in maintaining high-performance filtration [75]. The mechanistic impact of microplastic fouling on membrane structure and separation performance were tabulated in Table 2. Ultimately, microplastic-induced fouling does not merely slow down operations; it fundamentally alters the molecular environment of the membrane, requiring a focus on more resilient and fouling-resistant designs.

### 3.3. Cleaning and Antifouling Strategies

The operational integrity of polymeric membranes is frequently compromised by the accumulation of unwanted materials, a process that significantly reduces the flux of filtered liquids and forces systems to operate under higher, more costly pressures. In the context of membrane systems, implementing a proactive management strategy is not merely an engineering preference but an environmental necessity to extend equipment lifespan and minimize the chemical waste associated with frequent maintenance. By integrating sophisticated cleaning protocols with advanced surface modifications, it is possible to mitigate the economic and ecological footprints of these systems [76].

Mechanical intervention remains a cornerstone for removing established bio-waste and debris. Powered rotary brush systems are widely utilized, employing various materials such as nylon, polypropylene, and rubber [77]. The selection of brush stiffness is critical to effectiveness; for example, rubber brushes are recommended for harder epoxy surfaces, whereas nylon is often better suited for more delicate antifouling or fouling release coatings [78]. However, direct contact cleaning carries an inherent risk of creating surface scratches or irreversible damage, which can inadvertently provide new attachment sites for fouling organisms.

**Figure 1:**
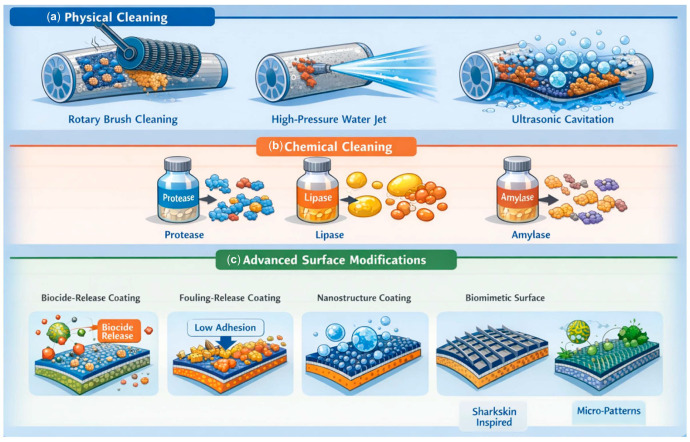
Integrated antifouling and cleaning strategies for maintaining the operational integrity of polymeric membranes: (**a**) physical cleaning, (**b**) chemical cleaning, and (**c**) advanced surface modifications.

To counter the risks of physical abrasion, non-contact technologies such as high-pressure water jets and cavitation cleaning have gained traction (Figure 1a). These systems use localized pressure changes to destroy biofouling without direct contact, thereby preserving the membrane’s surface topography [79]. For more delicate applications, ultrasonic antifouling systems offer a proactive solution by utilizing “acoustic cavitation”. High-frequency sound waves create microscopic bubbles that rupture the cells of fouling organisms before they can firmly attach [80]. This approach is particularly advantageous as it reduces the risk of environmental contamination by bio-wastes and microplastics typically associated with traditional scrubbing.

When physical methods are insufficient, chemical cleaning is employed to remove visible “soil,” defined as the removal of both organic and inorganic material (Figure 1b). Traditional detergents are often supplemented with enzymatic cleaners, which are prized for their ability to destabilize the extracellular polymeric substances (EPS) that provide structural integrity to biofilms [81]. Specific enzymes are selected to target different fouling components: proteases break down proteins, lipases target fats and grease, and amylases are utilized for starch-based soils. These biological agents improve detachment efficiency and represent a milder alternative to harsh chemicals that might degrade membrane polymers.

The most effective long-term strategy for preventing fouling is the engineering of the membrane surface itself (Figure 1c). Fouling control coatings (FCCs) generally fall into two categories: biocide-release and biocide-free technologies [82]. Self-polishing copolymers (SPCs) represent a chemically active approach where bioactive agents are gradually released through hydrolysis [83]. Conversely, fouling release coatings (FRCs), typically based on silicone or fluoropolymers, create ultra-smooth, low-friction surfaces that prevent organisms from sticking or allow them to be dislodged by simple liquid movement [84].

Nanotechnology has introduced transformative solutions, such as diamond-like carbon (DLC) nanostructure coatings. When applied to polyethersulfone (PES) membranes, DLC layers can increase salt separation from 64% to 98% and drastically improve pure water flux. These coatings reduce surface roughness, leading to a higher flux recovery rate and a significantly lower overall fouling rate. Furthermore, biomimetic surfaces, such as those inspired by sharkskin, use microscopic topography to increase the energy required for bacteria to colonize a surface, impeding attachment without toxic chemicals [85]. Finally, the development of self-healing silicone-based coatings provides a vital safeguard; these materials autonomously repair physical damage that would otherwise serve as primary sites for fouling accumulation.

### 3.4. Long-Term Operational Implications

Microplastics have become persistent residents in our wastewater treatment infrastructure. Their effective interception by membranes leads to a gradual but steady accumulation over years of operation. This buildup is more than a physical hurdle as it triggers complex biological and chemical shifts that threaten the long-term efficiency of systems like membrane bioreactors [86]. In the long term, we must consider how these particles change the environment for the microorganisms tasked with the heavy lifting of cleaning our water.

One of the most significant changes involves the increased secretion of extracellular polymeric substances and soluble microbial products. Long-term exposure to microplastics can stress these microbial communities, causing them to produce more of these sticky substances as a survival response or a reaction to cell damage [87]. These polymers act as the primary building blocks for biofouling, creating a thick and resilient layer on the membrane surface. As this layer develops, it accelerates the fouling process and makes it increasingly difficult to maintain a steady flow of clean water [88].

The biological health of the system also takes a substantial hit over extended operation periods. The accumulation of plastic particles tends to reduce the overall diversity and richness of the microbial community [89]. We see shifts where certain hardy bacteria like Clostridia become more abundant while others that are crucial for breaking down pollutants are suppressed. This biological imbalance can eventually compromise the entire treatment process, leading to a reduction in the degradation of organic pollutants [90]. There is also the issue of direct cytotoxicity, where these particles damage cell membranes and further hinder microbial activity.

The economic reality of these changes is found in the rising operational costs of the treatment facility. As fouling layers thicken, the system must work much harder to push water through the membrane. This requires higher operational pressures, which can lead to a jump in energy consumption by as much as 30 percent. Beyond the power bill, facilities face the rising cost of manual maintenance [91]. Frequent cleaning cycles using harsh chemicals become a necessity, which leads to more system downtime and higher labor costs.

These chemicals are not gentle on the equipment, and over several years, they begin to erode the membrane material itself. Perhaps the most daunting long-term implication is the shortened lifespan of the filtration membranes. Under the constant stress of microplastic fouling and aggressive chemical cleaning, a membrane that should last a decade might fail much sooner. Studies suggest that its functional life can be cut by 30 percent or even 50 percent. This forces a cycle of premature and expensive replacements that drain infrastructure budgets [92].

Additionally, the accumulation of microplastics impacts the physical characteristics of the sludge, making it harder to settle and more difficult to dewater. This complicates the final disposal process and adds another layer of logistical expense for the operator. While some research has shown that very high concentrations of microplastics might provide a temporary scouring effect that cleans the membrane surface, this is a double-edged sword [93]. The same physical rubbing that removes a fouling layer can also cause mechanical abrasion. Over time, this abrasion can thin the membrane or enlarge its pores, eventually allowing contaminants to slip through into the treated water. Ultimately, managing these permanent contaminants requires a shift toward more advanced and durable technologies to ensure that our water systems remain sustainable for the coming decades [94].

## 4. Advanced Approaches and Innovations

### 4.1. Functionalized and Smart Membranes for Selective Plastic Retention

**Figure 2:**
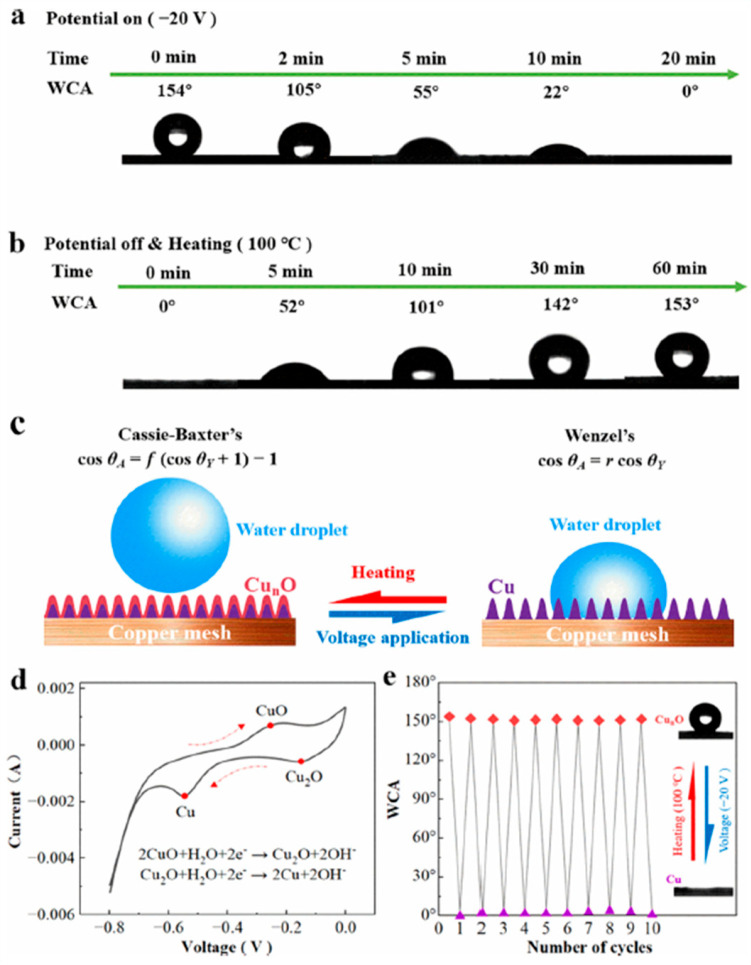
Reversible superwetting conversion of the as-prepared CM_e_ surface. (**a**) Evolution of the water contact angle and side-view images of a water droplet on the CM_e_ surface under an applied reduction potential of −20 V. (**b**) Behavior of the water droplet after switching off the applied potential and heating the membrane to 100 °C. (**c**) Schematic illustration of the complete reversible superwetting conversion process, where θ represents the water contact angle on an ideal smooth surface and a real rough surface, respectively. The roughness factor is denoted by *r*, while *f* represents the surface fraction of the solid–liquid interface. Y and A denote additional interfacial parameters as defined in the model. (**d**) Cyclic voltammetry (CV) profile of the electrodeposited copper mesh under applied external potential. (**e**) Water contact angle (WCA) variation over 10 cycles demonstrating reversible wettability transition. Reproduced from [98] under Copyright Clearance Center license, Copyright 2025, Publisher American Chemical Society.

The integration of smart materials into membrane technology marks a pivotal advancement in the selective retention and remediation of microplastics (MPs) [95]. Smart membranes (Figure 2) are designed to dynamically alter their chemical or physical properties in response to specific environmental stimuli—such as pH, temperature, light, and electrical fields; overcoming the fixed structural limitations of traditional systems [96]. These advanced approaches utilise stimuli-responsive motifs to adjust pore sizes, surface charge, or hydrophilicity on demand, enabling high-performance filtration and molecular sieving in complex wastewater environments [97].

A significant innovation in this field is the development of reversible superwetting transition membranes. For instance, researchers have engineered copper-based membranes that switch between superhydrophilic and superhydrophobic states through alternating electrical voltage and heat [98]. In its superhydrophilic state, the membrane forms a protective hydration layer through strong hydrogen bonding, which creates repulsion that effectively blocks foulants like poly(vinyl alcohol) (PVA), a common microplastic surrogate achieving a removal efficiency of 83.0%. When the membrane requires maintenance, it can be reverted to a superhydrophobic state via thermal treatment at 100 °C, facilitating the detachment of accumulated plastics through a “lotus effect” mechanism where water droplets roll off and carry away contaminants [99].

Bioinspired technologies further enhance selectivity by mimicking biological systems, such as fish gills or plant roots, to create intelligent “water gates” [95]. These gates, often composed of dual-responsive microgels loaded onto traditional nylon substrates, can sense specific environmental changes to switch from “open” to “closed” states [100]. Such mechanisms are being adapted for selective MP retention; for example, MXene-mangrove filled polylactic acid (PLA) membranes have demonstrated pH-responsive adsorption, achieving up to 99.0% rejection of polystyrene microplastics at pH 10 while simultaneously enhancing water permeability. Additionally, Polymer Inclusion Membranes (PIMs) provide extreme specificity by embedding task-specific carriers—such as ionic liquids or task-specific extractants—within a polymer matrix [101]. These carriers interact selectively and reversibly with target species, facilitating transport through cycles of complexation and decomplexation at ambient pressure.

Sustainability is also a primary driver in membrane functionalisation, with research focusing on upcycling polyolefin waste into high-performance membranes through acid-catalysed oxidation [102]. This process introduces oxygen moieties into the upcycled plastic structure, achieving a balance between superoleophilicity to trap hydrophobic contaminants and increased hydrophilicity to promote faster water passage. Ultimately, these innovations point toward a future where AI-assisted monitoring and smart functional materials work in tandem to provide real-time, adaptive remediation of plastic pollutants across global aquatic ecosystems [103,104].

### 4.2. Photocatalytic and Reactive Membranes for Degradation of MPs

The management of microplastic (MP) pollution is a critical environmental challenge, as traditional wastewater treatment plants (WWTPs) are not specifically designed to retain particles smaller than 20 μm. While physical separation methods such as membrane filtration can effectively isolate these pollutants, they are inherently non-destructive, merely transferring the plastics from the aqueous phase to a concentrated retentate or the membrane surface. To address this, current research is shifting toward Advanced Oxidation Processes (AOPs), particularly photocatalysis, to achieve the complete mineralisation of plastics into water and carbon dioxide.

A primary challenge in treating MPs is their low concentration within vast volumes of effluent, which makes direct treatment economically unfeasible. Recent innovations have proposed hybrid systems that use membranes to concentrate pollutants before subjecting them to a secondary degradation step [105]. For instance, a system combining microfiltration (MF), ultrafiltration (UF), or nanofiltration (NF) with TiO_2_-based photocatalysis has demonstrated significant potential in treating polyester fibres from laundry wastewater. In these configurations, nanofiltration (2 kDa CA membrane) has proven particularly effective, achieving a cleaning efficiency (CE%) of 98% and low irreversible fouling while capturing the smallest plastic particles. Subsequent photocatalytic treatment of the concentrated retentate using UV light and TiO_2_ can result in a 13.52% weight loss of polyester fibres within 48 h.

Photocatalytic degradation occurs through the generation of Reactive Oxygen Species (ROS), such as hydroxyl (•OH) and superoxide (O_2_•^−^) radicals, which attack and cleave the polymer backbones [106]. To overcome the limitation of pure TiO_2_, which is only active under UV light due to its 3.2 eV bandgap, researchers use doping and heterojunctions to enable visible light activity. Incorporating elements like Nitrogen (N) or Carbon (C) reduces the bandgap, allowing the material to harvest solar energy more effectively. For example, GO/N-TiO_2_ composites have shown a removal efficiency of 98.2% for PVC-NPs under visible light [107]. The integration of TiO_2_ with other semiconductors, such as g-C_3_N_4_ or iron-modified aerogels, creates a “Z-scheme” or “S-scheme” that prevents the recombination of photogenerated electrons and holes. Iron-modified TiO_2_ aerogels have been shown to accelerate the oxidative degradation of Polystyrene (PS) through combined photocatalytic and photo-Fenton processes [108].

Metal–Organic Frameworks (MOFs) represent an emerging class of reactive materials that can be integrated with polymers to create multifunctional membranes [109]. Certain MOFs, such as MIL-100(Fe) and HKUST-1, possess large specific surface areas and abundant active sites that enhance the adsorption and subsequent degradation of MPs. These “reactive platforms” can be tailored to cleave specific chemical bonds, such as C-O bonds in PET, potentially converting plastic waste into high-valued products like hydrogen or small organic molecules. Furthermore, waste plastics can be recycled as ligands to build these MOFs, supporting a circular economy model.

The efficacy of these reactive membranes is influenced by the polymer type, with a general degradation order of PS > Nylon 6 > PVC > PE > PP > PET. Smaller particles, such as NPs, typically degrade faster due to their higher surface-area-to-volume ratio. Despite this progress, several challenges remain, including irreversible membrane fouling, the potential toxicity of degradation metabolites, and the need for long-term stability in real-world wastewater conditions.

### 4.3. Coupling Membranes with Advanced Oxidation Processes (AOPs)

The integration of advanced oxidation processes (AOPs) with membrane filtration has emerged as a sophisticated approach to overcome the limitations of traditional water treatment, such as membrane fouling and the insufficient removal of refractory organic pollutants. This synergy provides a dual mechanism of physical retention and chemical degradation, which is particularly relevant for the elimination of persistent contaminants like microplastics and their associated organic additives.

#### 4.3.1. Catalytic Membranes and Pore Confinement

A major innovation in this field is the development of catalytic membranes that incorporate active sites directly into the membrane structure. Recent research has focused on single-atom catalytic membranes (SACMs), which utilise nearly 100% of their atomic density to activate oxidants like peroxymonosulfate (PMS) [110]. For instance, the creation of Fe–N3P1 active sites within ceramic membrane channels facilitates a “confined environment” that enriches both oxidants and pollutants [111]. This pore-confinement effect significantly reduces mass transfer resistance and enhances reaction kinetics, with some catalytic membranes achieving degradation rate constants several orders of magnitude higher than their powdered catalyst counterparts [112].

Similarly, one-dimensional carbon nanofiber membranes fabricated via electrospinning offer a hierarchical porous structure that exposes more active sites [113]. By doping these fibers with iron clusters, researchers have created robust filters that activate persulfates to degrade complex organic chains via radical (hydroxyl and sulfate radicals) and non-radical (singlet oxygen) pathways.

#### 4.3.2. Electrified Membrane Systems

Electrified membranes represent another frontier in AOP-membrane coupling. These systems often utilise the membrane as a cathode to facilitate in situ chemical reactions. A notable approach involves electrified reduced graphene oxide (rGO) membranes, where an external electric field maintains the catalytic activity by promoting a redox cycle between C–O and C=O functional groups [114]. This electrochemical regeneration prevents the oxidative inactivation of the carbon surface, ensuring long-term stability and efficient pollutant removal in complex water matrices [115].

Additionally, coupling membranes with in situ electrosynthesis using anthraquinone molecular catalysts has been proposed [116]. This method avoids the risks associated with the storage and transport of by generating it directly at the electrode-membrane interface, which can then be coupled with ozone (electro-peroxone) to degrade refractory phenolic compounds and potentially polymer fragments.

#### 4.3.3. Performance, Fouling, and Economic Perspectives

Coupled AOP-membrane systems offer a significant advantage in fouling control. The oxidative species generated at the membrane surface can degrade transformation by-products and organic foulants that typically block membrane pores. In large-scale pilot studies, coupling AOPs (such as photo-Fenton or UV/) with downstream filters like Granular Activated Carbon (GAC) has been shown to extend the operational lifetime of the system components and eliminate selective adsorption issues.

From an economic perspective, while coupled AOP-membrane systems often incur higher operational costs due to energy consumption (primarily for UV lamps or electrolysis), they provide a more sustainable quaternary treatment in the long term by reducing the frequency of membrane replacement and ensuring higher effluent quality [117]. Sensitivity analyses suggest that transitioning to renewable energy sources can reduce the global warming impact of these energy-intensive processes by up to 72% [118].

### 4.4. Green Chemistry Approaches in Sustainable Membrane Fabrication for Microplastic Removal

The integration of “Green Chemistry” into membrane fabrication marks a critical paradigm shift in water treatment, ensuring that the process of mitigating microplastic (MP) pollution does not inadvertently trigger secondary environmental harm through toxic manufacturing legacies or “pollution shifting.” While conventional membrane technologies are highly effective at capturing microplastics, they often rely on fossil-based polymers and aggressive, non-biodegradable solvents that can leach into the environment or generate their own microplastic litter upon degradation [119]. To address this, a “benign-by-design” approach has emerged, prioritizing the transition toward sustainable bio-based feedstocks such as cellulose, chitosan, lignin, and bio-polyamides. A notable technical innovation is the use of PA 6.9, a bio-based polyamide synthesized from plant or microalgae oil-derived azelaic acid; when processed into self-standing electrospun nonwovens, these membranes have demonstrated MP filtration efficiencies as high as 99.8% while maintaining superior mechanical stability and solvent resistance [120].

However, a critical perspective reveals that the sustainability of these materials is deeply tied to their fabrication process and ultimate lifecycle. The movement advocates for replacing hazardous organic solvents, such as N-methyl-2-pyrrolidone (NMP) or dimethylacetamide (DMAc) with greener alternatives like N-methylmorpholine-N-oxide (NMMO) or methyl lactate, and the adoption of solvent-free techniques like melt-spinning and 3D printing [121]. Technical reviews highlight that modifying the coagulation environment is key; for instance, cellulose membranes coagulated in an isobutanol bath rather than traditional water baths exhibit significantly enhanced porosity and flux, providing an eco-friendly route to high-performance filtration without toxic effluent [122]. Furthermore, the integration of biodegradable crosslinkers, such as citric acid, or the use of physical crosslinking methods like freeze-thawing, ensures that the membranes do not release toxic byproducts or persistent fragments at the end of their operational life.

### 4.5. Emerging Materials: Nanocomposites, Biomimetic, and Electro-Membranes

Recent innovations in membrane science focus on the integration of nanomaterials to overcome the inherent efficiency–flux trade-off and mitigate membrane fouling during the removal of microplastics (MPs). However, alongside these high-tech advancements, there is a significant shift toward utilizing renewable biopolymers like cellulose for membrane fabrication due to their low cost and environmental sustainability. Cellulose is an ideal candidate for this application as it is globally abundant, biodegradable, and possesses unique properties such as high mechanical strength and excellent biocompatibility [123].

Recent studies have demonstrated that the morphology and transport properties of cellulose membranes can be precisely tuned by varying the coagulation conditions, such as the nature and temperature of the precipitant [124]. For example, replacing traditional aqueous precipitation baths with alcohols like isobutanol allows for the formation of asymmetric, porous structures that can provide high permeability for filtration processes. These cellulose-based membranes have shown high efficiency in separating colloidal systems and could serve as an economically viable solution for large-scale microplastic separation [124].

Nanocomposite membranes, particularly those incorporating graphene oxide (GO), have demonstrated significant potential due to their high hydrophilicity, large surface area, and tunable surface charge. For instance, a GO-polyvinyl alcohol (PVA) composite membrane utilizes electrostatic repulsion between the negatively charged membrane surface and similarly charged plastic particles to separate high-density polyethylene (HDPE) microplastics with up to 95% efficiency [125]. Similarly, polyethersulfone (PES)-GO ultrafiltration membranes have been applied to treat river water, achieving a 91% reduction in MP pollutants [126]. Beyond traditional synthesis, laser bombardment can be used to tune the morphology of GO, creating a “labyrinth” structure with abundant defects and undulating wrinkles [127]. This structural modification enables a filtration flux of up to 3396 L m^−2^ h^−1^ bar^−1^, representing a significant enhancement compared to unirradiated membranes while maintaining approximately 99% filtration efficiency for various MPs [127].

Biomimetic membranes represent another frontier, drawing inspiration from natural biological transport systems to achieve high water permeability. Ceramic-based aquaporin (AQP) biomimetic membranes supported by anodic aluminium oxide (AAO) substrates leverage uniform cylindrical pores to facilitate the stable immobilisation of AQP vesicles [128]. Such systems have achieved high water flux (27.6 LMH) and superior membrane selectivity [128]. Furthermore, cryodesiccation (freeze-drying) has been introduced as a method to enhance the shelf-stability and transportability of these structures, allowing them to be stored in a dry state without compromising performance upon rehydration [129].

The integration of electro-membranes and hybrid electro-filtration systems offers an active approach to fouling control and small-particle separation. One proposed hybrid system combines physical filtration with ion concentration polarization (ICP), which creates an electric force barrier that arrests microplastics passing through a coarse filter lattice [130]. This approach achieves removal efficiencies exceeding 99.9% regardless of MP shape or size, with ultra-high fluxes of 10,000 L m^−2^ h^−1^ [130]. Additionally, electrified Ti_3_C_2_T_x_ MXene–SPES (sulfonated polyethersulfone) composite membranes have been investigated for MP separation in real hospital wastewater [131]. These electrified membranes operate in an intermittent ON/OFF voltage mode, generating hydrogen gas bubbles and in situ coagulants that disrupt fouling layers and aggregate MPs, resulting in over 90% rejection with minimal additional energy consumption.

### 4.6. Comparative Performance of Advanced Membrane Materials for Microplastic Removal

Electrostatic repulsion (*G_EL_*) plays a critical role in preventing microplastic adhesion to membrane surfaces. Graphene oxide (GO) membranes exhibit a highly negative zeta potential of approximately −200 mV, which generates a strong electrostatic repulsive barrier when interacting with negatively charged microplastics such as polystyrene (−25.7 mV), polyethylene (−12.5 mV), and polypropylene (−6.29 mV) [130]. This repulsion can be further enhanced under alkaline conditions; for instance, operating at pH 8 increases the negative charge density on both the membrane and microplastic surfaces, thereby strengthening electrostatic resistance to particle deposition [125]. In addition to electrostatic effects, acid–base interaction energy (*G_AB_*) significantly contributes to fouling resistance through surface hydrophilicity and hydration layer formation. Laser-modified GO membranes demonstrate a super-hydrophilic state, characterized by an underwater oil contact angle of 159.5°, which is 25.2° higher than that of pristine GO, thereby creating a substantial energetic barrier against hydrophobic plastic adhesion [127]. Surface roughness also strongly influences adhesion behavior; biomimetic membrane systems indicate that maintaining a surface roughness below 10 nm is crucial for minimizing fouling. For example, reducing the substrate roughness *R_q_* from 19.6 nm to 9.16 nm eliminates nanoscale surface valleys that otherwise act as high-energy trapping sites for microplastic fragments [128]. Beyond surface properties, the dynamic balance of forces during filtration governs particle rejection. The rejection threshold can be described by the equilibrium between drag velocity (*u_drag_*) and electrophoretic velocity (*u_ep_*); when *u_ep_* equals *u_drag_*, particles are effectively suspended away from the membrane surface, preventing the initiation of Lifshitz–van der Waals (*G_LW_*) or acid–base (*G_AB_*) adhesion mechanisms [130]. Furthermore, active fouling mitigation strategies such as electrified membrane systems have demonstrated significant improvements in operational performance. Intermittent voltage application can disrupt the formation of the cake layer through bubble-induced turbulence, resulting in a flux recovery ratio (*FRR*) of approximately 92%, compared to only about 38% under passive conditions, highlighting the effectiveness of electrically assisted fouling control [131]. Table 3 shows the comparative analysis such as surface morphology and zeta potential across innovative membrane materials.

## 5. Future Perspectives

### 5.1. Research Gaps in MP/NP Removal Mechanisms

**Figure 3:**
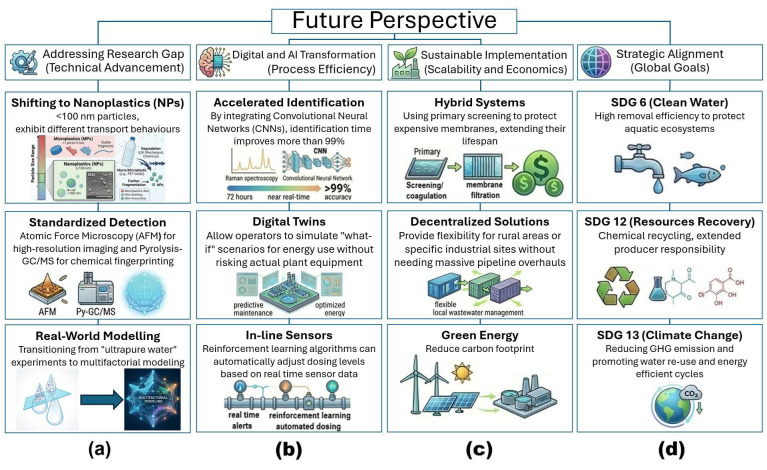
Future perspectives and strategic pathways for advancing membrane-based MPs removal.

The removal mechanisms of microplastics (MP) utilizing membrane filtration technologies are relatively well established (Figure 3a). However, the characterization and study of nanoplastics (NP), defined as plastic particles less than 100 nm in size, remains insufficiently explored, with studies indicating that only a minority explicitly focus on their removal. This gap is significant given the inherent differences in transport behaviors and bioavailability between MP and NP [132]. Moreover, treatment processes often inadvertently convert larger microplastics into nanoplastics, generating particles that current removal protocols may not effectively manage [133]. The differentiation between MPs and NPs is crucial, as their distinct ecological interactions and impacts necessitate tailored approaches [134,135].

In response to these challenges, future research should include: (a) the development of standardized detection methods for particles under 100 nm, incorporating technologies like pyrolysis gas chromatography/mass spectrometry and atomic force microscopy [136]; (b) computational modeling to study the molecular interactions between NPs and filtration membranes [137]; and (c) assessments of the fate and risks associated with concentrated NPs compared to dispersed forms [135,136].

Further complicating this scenario, most laboratory experiments utilize synthetic microplastics in ultrapure water, which contrasts starkly with the heterogeneous conditions encountered in real-world wastewater characterized by organic matter, temperature variabilities, and competing contaminants [138]. Evidence indicates that microplastics can enhance microbial biosynthetic activity, thereby increasing membrane fouling; however, this relationship is complex and can depend on factors such as calcium concentration and organic content present in wastewater [139]. This underscores the immediate necessity for multifactorial experiments that evaluate the influences of pH, ionic strength, natural organic matter, temperature, and microplastic loading on filtration dynamics [140]. Techniques such as quartz crystal microbalance and confocal laser scanning microscopy can significantly enhance our understanding of fouling behaviors in real-time settings [140].

### 5.2. Integration with Digital Monitoring and AI-Assisted Process Optimization

Current methodologies for quantifying microplastics can be exceptionally time-consuming, ranging from 4 to 72 h per sample, which poses barriers to effective adaptive management (Figure 3b) [141]. The integration of artificial intelligence (AI) with spectroscopic techniques could drastically accelerate the identification process of microplastics, with recent advancements showcasing classification accuracies potentially exceeding 95% for various polymer types [142]. Notably, the combination of Raman spectroscopy and machine learning has achieved accuracy levels above 99% [143]. Moreover, the application of computer vision techniques utilizing convolutional neural networks shows promise for segmenting microplastics and has proven effective when trained with extensive datasets [144].

Furthermore, in-line sensors facilitate continuous monitoring of microplastic concentrations at different stages of treatment, delivering real-time alerts to optimize operational responses when loads exceed treatment capacities [145]. The concept of digital twins—virtual replicas that update with real-time data—emerges as a transformative strategy for predictive maintenance, with studies advocating the integration of data collection tools such as pressure transducers and flow meters to enhance performance predictions [146]. The anticipated outcomes of these digital advancements include reduced energy consumption, prolonged membrane longevity, decreased chemical cleaning demands, and improved water recovery rates, all contributing to overarching sustainability goals.

Reinforcement learning frameworks can promote holistic optimization across treatment systems by balancing efficient microplastic removal and resource conservation. Simulations exploring tailored coagulant dosing strategies have produced notable outcomes, highlighting significant energy savings alongside improved effluent quality [147].

### 5.3. Pathways Toward Sustainable Implementation in Full-Scale Plants

Retrofitting existing wastewater treatment plants with advanced filtration technologies requires substantial financial investment (Figure 3c). However, the economic rationale for such upgrades can be justified when framed within pollution prevention strategies, as the OECD estimates annual costs associated with plastic pollution to be significantly high [148]. Hybrid treatment systems, utilizing sequential processes such as primary screening and coagulation-flocculation, can effectively lower operational expenses while boosting removal efficiencies [149].

The incorporation of renewable energy sources is critical in mitigating both financial costs and the carbon footprint associated with wastewater treatment operations. Successful implementations, such as Singapore’s wastewater treatment initiatives, exemplify the potential for reduced energy expenses, lower carbon emissions, and improved resource recovery through renewable energy integration [135]. Notably, containerized membrane units present a flexible alternative for decentralized wastewater treatment, offering efficient management for recovery and alleviating burdens on centralized systems [150].

### 5.4. Alignment with UN Sustainable Development Goals

#### 5.4.1. SDG 6 (Clean Water and Sanitation)

By implementing membrane technology across a significant portion of the global wastewater treatment infrastructure, one could achieve removal efficiencies ranging 90% to 99%, potentially preventing trillions of microplastics from infiltrating ecosystems annually (Figure 3d) [151]. Case studies from countries like Singapore demonstrate the scalability of such technologies, thus playing a pivotal role in advancing water reuse and overall resource efficiency [135].

#### 5.4.2. SDG 12 (Responsible Consumption and Production)

Pertinent to SDG 12, there are emerging opportunities for secondary resource recovery through innovative chemical recycling processes (Figure 3d). Advances in methods such as pyrolysis suggest significant reductions in greenhouse gas emissions compared to conventional production processes [152]. Additionally, implementing Extended Producer Responsibility models can lend financial support to enhance wastewater treatment frameworks, fostering sustainable practices [153].

#### 5.4.3. SDG 13 (Climate Action)

Given that plastic manufacturing is a major driver of global greenhouse gas emissions, recycling initiatives targeting nanoplastics hold promise for emission mitigation and resource conservation (Figure 3d). The development of water reuse practices via advanced membrane technologies can lead to substantial reductions in energy utilization tied to freshwater supply systems, embodying sustainability across environmental, economic, and social dimensions [154].

#### 5.4.4. Cross-Cutting Synergies

The deployment of microplastic removal technologies through membranes supports multiple Sustainable Development Goals, particularly in health by reducing human exposure to microplastics found in various biological studies. Successful initiatives also align with conservation efforts for marine ecosystems, fostering innovative infrastructure and promoting collaborative approaches to tackle plastic pollution challenges.

## 6. Conclusions

In summary, membrane technologies represent a superior alternative to conventional wastewater treatment, offering high-efficiency retention of MPs. However, several critical bottlenecks currently prevent these technologies from becoming the universal standard for MP removal. Physically, the industrial scalability of membranes remains hindered by high energy demands and complex fouling mechanisms. While the transition from traditional polymers to next-generation hybrid structures, such as MXene-incorporated thin-film nanocomposites offers a pathway to mitigate these issues through enhanced hydrophilicity and tunable transport channels, a more systemic bottleneck exists: the lack of standardized analytical methods. Currently, the absence of uniformity in how microplastic removal efficiency is quantified and reported across different studies makes it difficult to benchmark performance or compare the efficacy of different membrane materials. Discrepancies in sampling volumes, detection limits, and the characterization of particle morphology (fibers vs. fragments) create a fragmented landscape of data. Therefore, the development of standardized protocols for the identification and quantification of microplastics is a primary future research direction. Establishing these benchmarks is essential for validating the performance of advanced hybrid membranes and ensuring that results are reproducible and comparable on a global scale. By the integration of advanced materials with digital transformation, such as AI-assisted monitoring, the cost of membrane technology in wastewater management will be drastically reduced. Ultimately, the transition to the membrane-based wastewater management provides a crucial pathway in achieving the United Nations Sustainable Development Goals, ensuring global water security through the mitigation of microplastic pollution in our water.

## Figures and Tables

**Table 1:** Comparative analysis of membrane filtration processes for the removal of microplastics (MPs) and nanoplastics (NPs).

Membrane Process	Pore Size	Primary Removal Mechanisms	MPs/NPs Removal Efficiency	Key Advantages	Major Limitations
Microfiltration (MF)	0.1–10 µm	Size exclusion, cake layer formation, hydrodynamic interactions	>90% for particles > 10 µm	Low energy, high flux, excellent pretreatment	Limited NP retention; susceptible to surface fouling
Ultrafiltration (UF)	1–100 nm	Size exclusion, electrostatic interactions	>90% for NPs (<500 nm)	High-quality effluent; removes sub-micron scale particles	Internal pore blocking; requires frequent cleaning
Nanofiltration (NF)	1–10 nm	Size exclusion, electrostatic repulsion	>90% for NPs and dissolved organics	Concurrent removal of ions and nanoplastics	High operating pressure; complex concentrate disposal
Reverse Osmosis (RO)	Non-porous	Solution–diffusion	>99% for all plastic size fractions	Highest rejection reliability for potable reuse	High energy consumption; requires intensive pretreatment

**Table 2:** Mechanistic impact of microplastic fouling on membrane structure and separation performance.

Fouling Stage	Physical/Chemical Mechanism	Structural Impact on Membrane	Effect on Permeability	Effect on Selectivity	Long-Term Consequence
Initial Deposition	Microplastic particle adsorption and pore blocking	Surface coverage; partial pore occlusion	↓ Moderate flux decline	Slight change	Increased hydraulic resistance
Cake Layer Formation	Accumulation of microplastics + EPS	Formation of external mass transfer barrier	↓↓ Significant permeability loss	Possible artificial selectivity increase (temporary sieving effect)	Higher operating pressure required
Compaction Under Pressure	Compression of cake layer and polymer matrix	Structural deformation; densification or local pore distortion	Further ↓ flux	Reduced precision in size exclusion	Increased energy consumption
Internal Matrix Deformation	Stress-induced alteration of Free Volume Elements (FVEs)	Redistribution/enlargement of FVEs (MD-predicted)	Variable (may ↑ locally)	↓ Selectivity due to widened transport pathways	Shift toward Robeson trade-off limit
Repeated Chemical Cleaning	Polymer chain scission and aging	Permanent FVE instability; microcracks	Gradual irreversible decline	Loss of separation consistency	Shortened membrane lifespan

↓—decrease; ↓↓—decrease; ↑—increase.

**Table 3:** Comparative Analysis across Innovative Membrane Materials.

Membrane Material	Surface Charge (Zeta Potential)	Contact Angle (WCA/UOCA)	Surface Roughness (*R_q_*)	Resulting Rejection Rate (%)	Interaction Energy/Mechanism Focus
Graphene Oxide (GO) [125]	−200 mV	58.52°	High (Defects and wrinkled)	98.48% (at 1 μm)	High *G_EL_* repulsion against PS/PE particles
GO-PVA Composite [125]	−100 mV	71.28°	High (coarse topography along bumpy surface)	95% (HDPE)	Acetalization reduces *G_EL_* barrier compared to pure GO
Laser Modified GO [127]	N/A	159.5° (UOCA)	High (Wrinkled)	~99% (PVC/PET)	Super-hydrophilicity creates dominant *G_AB_* hydration barrier
AAO-Aquaporin (AQP) [128]	N/A	N/A	9.16 nm	0.11 gL^−1^ (SRSF)	Low *R_q_* minimizes physical entrapment and physical fouling
Electrified MXene-SPES [131]	Negative	Hydrophilic	High (Discrete circular flocs and scattered deposits)	>90% (NP)	Intermittent voltage disrupts *G_LW_* adhesion via bubble turbulence
PES-GO Membrane [126]	N/A	68°	N/A	91% (River MPs)	Functional groups modify pore structure to reduce *G_LW_* adhesion

WCA—water contact angle; UOCA—under oil contact angle.

## Data Availability

No new data were created or analyzed in this study.
